# Differences in self-reported weekend catch up sleep between children and adolescents with and without primary hypertension

**DOI:** 10.1186/s40885-018-0092-6

**Published:** 2018-04-05

**Authors:** Neena Gupta, Louise Maranda, Rakesh Gupta

**Affiliations:** 1 University of Massachusetts Children’s Medical Center, Division of Pediatric Nephrology, 55 Lake Avenue North, Benedict Bldg, A2 210, Worcester, MA 01655 USA; 2 University of Massachusetts Memorial Medical Center, Quantitative Health Sciences, Worcester, MA 01655 USA; 3 PrimaCare Sleep Center, Somerset, MA 02726 USA

**Keywords:** Sleep duration, Blood pressure, Hypertension, Children, Adolescents

## Abstract

**Background:**

The data on the association of sleep duration and blood pressure in the pediatric age group have been mixed and most studies have focused on weekday sleep duration. The purpose of this study was to compare the weekday and weekend sleep patterns between children and adolescents with newly diagnosed primary hypertension and a normotensive control group.

**Methods:**

Children and adolescents from a pediatric nephrology clinic, aged 6-18 years with newly diagnosed primary hypertension were compared to an age and sex matched normotensive control group from a general pediatric clinic. The questions about bed time and getting out of bed times from the Pediatric Sleep Questionnaire (PSQ) were used to obtain weekday and weekend bed time, getting out of bed time and sleep duration. The Pediatric Daytime Sleepiness Scale (PDSS) was used to assess subjective sleepiness.

**Results:**

In both groups of 60 subjects each, weekday total sleep time was similar. Subjects in both groups went to bed later and woke up later on the weekends. However, in the hypertensive group, weekend getting out of the bed time was earlier (8:52 AM ±93 min vs. 9:36 AM ±88 min, *p* = 0.013) and weekend catchup sleep was about 40 min less (62.8 ± 85.5 vs. 102.7 ± 84.9, *p* = 0.035). Hypertensive children perceived less subjective sleepiness (PDSS scores 8.28 ± 4.88 vs. 10.63 ± 5.41, *p* = 0.007). The *p* values were calculated after adjusting for body mass index (BMI), race, daytime nap, caffeine use, sleep related breathing disorder (SRBD) scale and periodic limb movement of sleep (PLMS) scale subcomponents of the PSQ.

**Conclusions:**

Hypertensive children obtained less weekend catch up sleep and reported less subjective sleepiness compared to the control group. More weekend sleep may potentially mitigate the effect of weekday sleep deprivation on blood pressure.

## Background

It is important to understand the factors affecting blood pressure (BP) in children as elevated BP in childhood predicts BP in adulthood [1]. Childhood hypertension has been shown to cause target organ damage like left ventricular hypertrophy, increased carotid intimal thickness and microalbuminuria that are known to predict future cardiovascular events [2].

Etiology of hypertension in pediatric population is multifactorial and has been attributed to genetic, renal, endocrine, excess weight, sedentary lifestyle and sleep related factors. Recent interest in the relationship between sleep and BP comes at a time when the self-reported sleep duration in adolescents has been declining over last 20 years [3] and remains significantly below the recommended amount of sleep [4].

Commonly proposed mechanisms for increase in BP due to insufficient sleep include sympathetic system over-activity, higher cortisol levels and endothelial dysfunction [5–7]. One night of sleep deprivation can also increase arterial stiffness [8]. Other metabolic effects of insufficient sleep like insulin resistance, increased inflammatory markers, and changes in appetite with a preference for salty and sweet foods may also impact blood pressure indirectly [5, 6]. Most of these studies have assessed the effects of short term sleep deprivation only.

Data on the effect of habitual sleep duration on BP in pediatric age group are limited and they show mixed results [9]. Some studies show an association between shorter sleep duration and BP or hypertension status [10–13], while others have found no relationship or the relationship was not significant after adjusting for covariates [14, 15] or found mixed results for different age groups [16]. Still other studies have reported an association of long sleep duration with higher odds of high blood pressure in females [17].

Most of the pediatric studies evaluating the association between sleep duration and BP have been in a cohort of normal children, have used different measures of sleep duration, and different cut offs for “short sleep duration” [10–17]. Some studies have estimated the sleep duration by averaging self-reported sleep time [10] or actigraphic sleep duration [11] over 7 days, some have used 1 d actigraphic sleep duration on a school day [13], some have used the self-reported sleep duration on school days only [17], some have used the 3 to 5 day actigraphic sleep duration on school days only [14] and others have used self-reported sleep duration from a question about “usual sleep duration” without reference to any day of the week [12, 15, 16]. However, the typical sleep pattern in school children in the United States is that of less sleep on weekdays and catch up on weekends; primarily by waking up later [18]. The main driver for such behavior is the need of waking up early for school on weekdays in the face of a biologically delayed circadian sleep wake rhythm at this age. It is a common professional recommendation that teenagers should not sleep in on weekends [19] to minimize “jet lag” but there is very limited evaluation of the effect of sleeping in on weekends on BP. A survey of Korean middle aged adults showed that the weekend catchup sleep was associated with lower prevalence of hypertension [20]. These limited data suggest that the contribution of weekend catch up sleep is not fully captured by the sleep duration averaged over 7 days or measured only on weekdays as has been done in most studies.

In this study, we compared hypertensive children with a group of age and sex matched normotensive children with respect to weekday and weekend sleep parameters such as sleep duration, bedtime, out of bed time and weekend-weekday sleep difference. We hypothesized that the hypertensive group would obtain less catch up sleep on the weekends.

## Methods

**Fig. 1:**
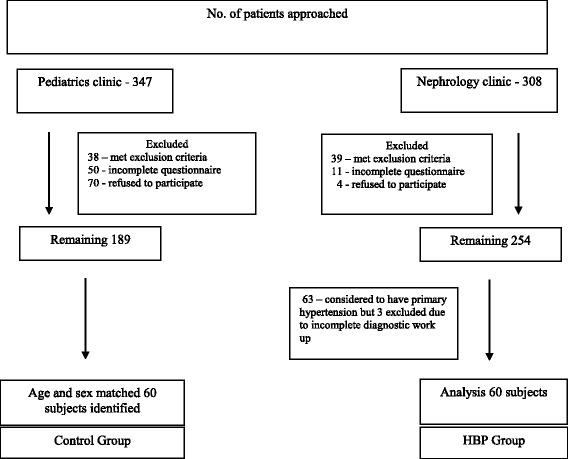
Flow chart for patient selection

Subjects for this study were identified from a cohort of subjects recruited from the pediatric nephrology clinic and a control group from the general pediatric clinic between Jan 2013 to Jan 2016 to study the prevalence of sleep problems in the pediatric nephrology patients (Fig. 1). From the pediatric nephrology patients, a subset of 63 children diagnosed with new onset primary hypertension (HBP group) was the group of interest for this study of which 3 patients were excluded due to incomplete diagnostic work up. As age and sex have a large effect on the BP in children, an age (matched to < 3 months difference) and sex matched control group of 60 normotensive children was identified from the general pediatric clinic for the robust control of these confounders. The study was approved by our Institutional Review Board.

Patients between the ages of 6-18 years were approached on the days when trained study personnel (nurse practitioner and medical students) were available. At the time of consenting, both, the guardian and the patient completed the sleep questionnaires together. Inclusion criteria required that the Body Mass Index (BMI) and clinic BP data be available in the medical record for both groups within 6 months. Exclusion criteria for both groups were: use of antihypertensive medications, any severe neurologic disorders (brain malformations, mental retardation, and cerebral palsy), or other severe co-morbidities (e.g. uncontrolled asthma, diabetes, chronic pain, symptomatic heart failure). For the HBP group, only children with newly diagnosed primary hypertension were included.

Demographics, BMI, BP and co-morbidities were extracted from the medical records. BMI categories were based on the age and sex specific percentiles defined by the US Centers for Disease Control and Prevention [21]. BMI < 85th percentile was considered as normal weight, ≥ 85th percentile but <95th percentile was considered as overweight and ≥ 95th percentile was considered as obese. BP was measured in the clinic using Welch Allyn Masimo SET Spot vital sign LXi instrument (model number 45MT0) by trained personnel. An average of all BP readings was taken when there were multiple BP readings available in one sitting in patients from the control group. In the HBP group, a physician repeated the BP measurement following the recommendations of the National High Blood Pressure Education Program Working Group on High Blood Pressure in Children and Adolescents to confirm HBP [22]. The patients were considered normotensive if systolic blood pressure (SBP) and/or diastolic blood pressure (DBP) was <90th percentile for age, sex and height, pre-hypertensive if SBP and/or DBP was ≥90th percentile but <95th percentile and hypertensive if SBP and/or DBP ≥ 95th percentile [22].

Questionnaires included the Pediatric Sleep Questionnaire (PSQ) [23] and the Pediatric Daytime Sleepiness Scale (PDSS) [24]. The PSQ is a validated instrument designed to assess various sleep symptoms. Sleep related breathing disorder (SRBD, most common form of SRBD being obstructive sleep apnea) and periodic leg movements during sleep (PLMS) scores were calculated using the SRBD [23] and PLMS [25] subscales of PSQ and a score of > 0.33 was considered as an indicator of higher likelihood of the respective disorder [23, 25]. Weekday and weekend sleep duration was calculated from response to the following PSQ questions: “What time does your child usually go to bed during week (during school days)?”, “what time does your child usually go to bed on weekend or vacation (when you are not in school)?”, “what time does your child usually get out of bed on weekday mornings (during school days)?” and “what time does your child usually get out of bed on weekend or vacation mornings (when you are not in school)?” Weekend catch up sleep was calculated as the difference between weekend and weekday sleep duration. Bedtime and out of bed time were converted to decimal format for statistical analysis. The PDSS is a validated instrument for assessing daytime sleepiness in school age children with a score range of 0-32. A higher PDSS score indicates more sleepiness. Use of caffeinated drinks and daytime nap was identified (as yes or no) from the responses to the questions in the PSQ.

### Statistical analysis

The final sample included 60 patients in each group (see Fig. 1). Continuous variables were compared between the two groups using independent sample t-tests and categorical variables were compared using chi-square tests. ANOVA was used to adjust for Body Mass Index-Standard Deviation Score (BMI-SDS), race, daytime nap, caffeine use, sleep related breathing disorder (SRBD) scale and Periodic limb movement in sleep (PLMS) scale subcomponents of PSQ. Linear regression and Pearson correlation coefficient (*p*-value calculated for a uni-directional hypothesis predicting less sleep time to be associated with more sleepiness) were used to assess the relationship of the sleep duration to PDSS scores. IBM SPSS Statistical software package 24 was used for these calculations.

## Results

### Subject characteristics

Demographic and anthropometric data are presented in Table 1. Of the 60 subjects in each group, 70% were boys and the mean age was 14.2 ± 3.3 years. As expected, BMI-SDS, SBP and DBP were significantly higher in the HBP group. Normal weight, overweight, and obesity were noted in 21 (35%), 11 (18.3%) and 28 (46.6%) in the HBP group and 45 (75%), 6 (10%), and 9 (15%) in the control group respectively. There were no significant differences between the two groups in the racial distribution, the proportion of the subjects taking caffeine, daytime nap, and abnormal SRBD and PLMS subscale scores from the PSQ. Racial composition of the HBP group was: white/not Hispanic 35 (58%), Hispanic 15 (25%), African-American 3 (5%), Asian-American 2 (3%), other 5 (8%) and in the control group was: white/not Hispanic 37 (62%), Hispanic 10 (17%), African-American 6 (10%), Asian-American 4 (7%), other 3 (5%).

**Table 1:** Baseline and sleep parameters

	HBP group (mean ± SD or %)	Control group (mean ± SD or %)	*P* value	*P* value adjusted for Race, Caffeine use, BMI-SDS, Daytime Nap, SRBD scale and PLMS scale
N	60	60		
Age (years)	14.2 ± 3.3	14.2 ± 3.3	0.96	
Gender (Male)	70	70	0.70	
Race (whites)	58	62	0.371	
BMI	25.8 ± 5.5	21.9 ± 4.2	< 0.00	
BMI- SDS	1.26 ± 0.99	0.55 ± 0.85	< 0.00	
BMI Percentile	82 ± 24	66 ± 23	< 0.00	
SBP mm Hg	131.5 ± 13.8	112.7 ± 9.6	< 0.00	
DBP mm Hg	79.4 ± 7.8	71.3 ± 6.7	< 0.00	
SRBD > 0.33	18 (30)	12 (20)	0.31	
PLMS > 0.33	20 (33.3)	17 (28.3)	0.64	
WD Bedtime (in decimal) (hour:min)	9.70 ± 1.02 9:42 PM	9.78 ± 0.99 9:47 PM	0.65	0.918
WE Bedtime (in decimal) (hour:min)	10.97 ± 1.34 10:59 PM	11.25 ± 1.40 11:15 PM	0.28	0.209
WD OOB time (in decimal) (hour:min)	6.55 ± 0.88 6:33 AM	6.47 ± 64 6:28 AM	0.62	0.526
WE OOB time (in decimal) (hour:min)	8.86 ± 1.55 8:52 AM	9.6 ± 1.46 9:36 AM	0.007	0.013
WD TST (minutes)	530.9 ± 74.9	519.6 ± 76.6	0.42	0.530
WE TST (minutes)	593.7 ± 79.1	622.3 ± 80.9	0.053	0.081
WE-WD TST (minutes)	62.8 ± 85.5	102.7 ± 84.9	0.012	0.035
PDSS total	8.28 ± 4.88	10.63 ± 5.41	0.014	0.007
Daytime nap - Yes	15 (25)	7 (11)	0.060	
Caffeine users	16 (26.6)	22 (36.6)	0.33	

*BMI* Body mass index, *BMI-SDS* body mass index standard deviation score, *SBP* systolic blood pressure, *DBP* diastolic blood pressure, *SRBD* sleep related breathing disorder scale score, *PLMS* periodic limb movements in sleep scale score, *WD Bedtime* weekday going to bed time, *WE Bedtime* weekend going to bed time, *WD OOB* week day getting out of bed time, *WE OOB* weekend getting out of bed time, *WD TST* weekday total sleep time, *WE TST* weekend total sleep time, *WE-WD TST* Weekend catchup sleep time calculated as WE TST minus WD TST, *PDSS* pediatric daytime sleepiness scale

### Sleep patterns

See Table 1 for details and the parameters used to calculate adjusted *p* value. In our local area, school hours are approximately from 7:10-8:15 AM to 1:45-3:00 PM. On weekdays, going to bed and getting out of the bed times were similar in both the groups resulting in similar mean weekday total sleep times (in minutes, 530.9 ± 74.9 in the HBP group vs. 519.6 ± 76.6 in controls, *p* = 0.42 and after adjustment 0.53). Weekend total sleep time tended to be longer in control subjects (in minutes, 622.3 ± 80.9 vs. 593.7 ± 79.1, *p* = 0.053 and after adjustment 0.081). The weekend bedtime was not statistically different between the groups. Subjects in both groups went to bed later on weekends as compared to weekdays. Compared to weekdays, weekend getting out of bedtime was also later in both groups. However, subjects in the control group woke up later than the subjects in the HBP group on weekends (9:36 AM ±88 min vs. 8:52 AM ±93 min, *p* = 0.007 and after adjustment 0.013). As a result, children in HBP group woke up 2 h and 19 min later on the weekend as compared to weekdays and had 62.8 min of weekend catchup sleep whereas those in the control group woke up 3 h and 8 min later on the weekend and had 102.7 min of weekend catchup sleep. Thus, the weekend catch up sleep in control group was about 40 min longer and significantly more than in the HBP group (102.7 ± 84.9 vs. 62.8 ± 85.5 min, *p* = 0.012 and after adjustment 0.035).

### Subjective sleepiness

**Fig. 2:**
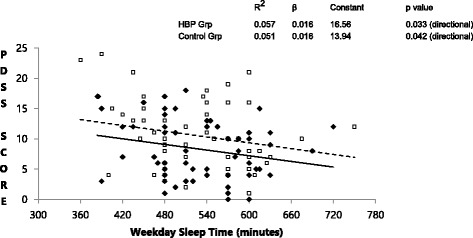
Relationship between weekday sleep time and subjective sleepiness. Weekday sleep time shows an inverse relationship with subjective sleepiness (PDSS score - pediatric daytime sleepiness scale) in both groups. The parallel but lower regression line in the HBP group (High blood pressure group) suggests qualitatively similar but quantitatively lower response to weekday sleep deprivation. (HBP group – solid diamonds, solid line; Control group – open squares, dashed line)

**Fig. 3:**
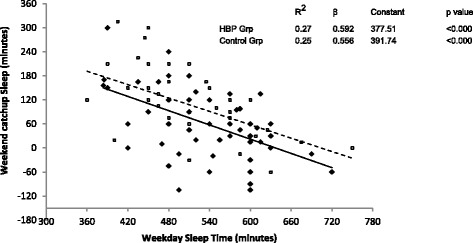
Relationship between weekday sleep time and weekend catchup sleep time. Weekday sleep time shows an inverse relationship with weekend catchup sleep time in both groups. The almost parallel but lower regression line in the HBP group (High blood pressure group) suggests qualitatively similar but quantitatively lower response. Many more children in HBP group have a negative weekend catch up sleep time. (HBP group – solid diamonds, solid line; Control group – open squares, dashed line)

In both groups, subjective sleepiness as measured by PDSS was inversely correlated with the weekday sleep time (Fig. 2) and weekday sleep time was inversely correlated with the weekend catch up sleep time (Fig. 3). This suggests that in both groups, children getting less sleep on the weekdays feel sleepier and children who feel sleepier obtained more catch up sleep on the weekend. However, when comparing the two groups, subjects in HBP group perceived significantly less subjective sleepiness (PDSS scores 8.28 ± 4.88 vs. 10.63 ± 5.41, *p* = 0.014 and after adjustment 0.007) even though there was no significant difference in the weekday sleep time between the two groups (Table 1). The slope for the increase in sleepiness and resultant catch up sleep in response to weekday sleep duration was same in both groups but the magnitude of this response was lower in HBP group (the regression lines in the HBP group were lower in magnitude but parallel to control group, Figs. 2 and 3).

## Discussion

In this study, we compared the sleep parameters (sleep duration, sleep timing and subjective daytime sleepiness) in a group of newly diagnosed hypertensive children and adolescents with an age and sex matched normotensive control group. While the sleep parameters on weekdays were similar between the groups, major findings from our study are that the subjects in the HBP group woke up earlier on weekends and obtained less catch up sleep on weekends compared to the normotensive control group. The HBP group also reported less subjective sleepiness compared to the control group. These significant differences in the sleep parameters persisted after adjustments (for BMI-SDS, race, daytime nap, caffeine use, SRBD scale and PLMS scale) in our study suggesting an independent association of weekend catch up sleep and BP.

Previous studies in children and adolescents looking at the association of sleep duration on BP have produced inconsistent results. Au et al. and Mezick et al. reported an association between shorter sleep (average of 7 days) and higher BP [10, 11]. On the other hand, Paciencia et al. found that the odds of high BP (> 90th percentile) were increased in female adolescents sleeping > 9.5 h per night (measured on week days only) compared to those sleeping < 8.5 h nightly [17]. Javaheri et al. examined sleep duration on school days and found short sleep duration to be associated with higher odds of prehypertension but the association was not significant after adjustment for sex, BMI and socioeconomic status [14]. Peach et al. showed that in 6th grade children, school-night sleep duration, weekend sleep duration and daytime sleepiness directly predicted BMI and indirectly predicted risk for hypertension via association with BMI [26]. Meininger et al. also found an inverse relationship between sleep duration and BP by measuring sleep duration and Ambulatory Blood Pressure Monitor (ABPM) together on a school day [13]. The role of weekend catch up sleep was not captured in any of these studies due to their different study designs.

Our study differs from these studies as we assessed weekday and weekend sleep separately. In contrast to other studies that have looked at the association between sleep duration and blood pressure in a single cohort, we compared sleep parameters of a newly diagnosed hypertensive group to an age and sex matched normotensive group. As all school going children are forced to conform to similar schedules on weekdays; it is understandable that the weekday bed time, getting out of bed time and total sleep time were similar in both the HBP and the normotensive groups. Based on the “2006 sleep in America Poll”, self-reported weekday bed time and out of bedtime for 7th, 8th and 9th graders were 2152, 2153, 2215 and 0635, 0636, 0628 h respectively and weekend bed time and out of bed time were 2305, 2326, 2353 and 0912, 0921, 0954 h respectively [18]. In our study which also used self-reported questionnaire data, the overall timings are very similar to the national data. The weekday bed time and out of bedtime means were 2142 and 0633 h for HBP group and 2147 and 0628 h for control group respectively. Weekend bed time and out of bed time were 2259 and 0852 h in HBP group and 2315 and 0936 h for control group respectively. The weekend out of bed time in control group was closer to the national average but it was earlier in HBP group. There was a mean difference of 44 min in our study in weekend out of bed time between HBP and control group.

There is a circadian phase delay with maturation of the biological regulation of sleep in adolescents which is responsible for their normal tendency to go to bed late and get out of bed late [18]. Besides this, psychosocial factors like social networking, pressure to be a high achiever, availability and use of new technology, also contribute to delay in going to sleep time although the need for total sleep time remains constant [18]. Wake up time could also be determined by external forces, like early school start times, to watch favorite television shows or athletic commitments. Students tend to wake up late on weekends due to both phase delay and to catch up on sleep debt accumulated on weekdays [18]. It is likely that the sleep pattern on weekend has better circadian alignment to their natural underlying rhythm whereas on weekdays, the sleep pattern is less aligned with their natural circadian rhythm. Students who wake up earlier on weekend may not get the full benefit of this week end catch up sleep. There are limited number of studies looking at the relationship between weekend catch up sleep and BP. A Korean survey of middle aged adults showed that more weekend catch up sleep was associated with lower risk of prevalent hypertension [20]. Studying this association in children may be more relevant to understanding the relationship between weekend catch up sleep and blood pressure because in adult studies, sleep duration is measured many years after the development of hypertension. Race has also been suggested as a factor affecting sleep duration and bedtimes [27]. Minorities comprised 40% of our subjects. The differences in sleep parameters between hypertensive and normotensive subjects remained significant after adjustment for race.

In this study, the control group reported more subjective sleepiness even though they had same weekday sleep time and longer weekend sleep time as compared to the HBP group. The weekday sleep duration was inversely correlated to subjective sleepiness in both groups confirming that both groups respond to sleep loss qualitatively in the same way but children in the hypertensive group seem to have lower magnitude of subjective sleepiness (Figs. 2 and 3). Also, weekday total sleep time was inversely proportional to the weekend catch up sleep in both groups but HBP group had comparatively less weekend catch up sleep. Several explanations are possible for this pattern. It is possible that some individuals have learned behaviorally to ignore or become desensitized to the sensation of sleepiness due to competing priorities. In our clinical practice, we have observed that some children wake up on weekend in time to watch their favorite television shows. It is also known that the ability to feel sleepiness (subjective sleepiness) is an intrinsic biological trait [28] and individuals have differential vulnerability to sleep loss [29]. Higher level of subjective sleepiness in the control group may either allow or force them to obtain more catch-up sleep on weekends. The dissociation between subjective sleepiness and physiological impairment due to insufficient sleep has been confirmed for some neurological parameters. Cognitive impairment [30, 31] and abnormalities on fMRI (functional Magnetic Resonance Imaging) of the brain [32] have been demonstrated even in short sleepers who may not report feeling sleepy.

Our study has several strengths. We have assessed weekday and weekend sleep duration separately. Our age and sex matched controls allowed for a more robust control of these confounders as these factors have a very large impact on BP in children and adolescents. We also confirmed the diagnosis of hypertension by ambulatory BP measurement. We compared sleep parameters of a group of children with high BP to a control group whereas, in other studies, the “association or relationship” of sleep duration to BP was evaluated in a single cohort of subjects. Confounding from sleep disorders such as obstructive sleep apnea or PLMS was addressed by adjusting for the SRBD and PLMS scale scores [23, 25].

At the same time, our study has some limitations. Subjects were recruited on days when study personnel were available. Although it was not a random sample, we do not suspect any systematic bias in the patient recruitment. As self-reported sleep time was calculated from response to questions, our data could be described as “nocturnal rest time” rather than “sleep time”. Thus the actual sleep time is likely to be less than the calculated sleep time in our subjects. The large magnitude of weekend catch up sleep in both groups is an indication of insufficient sleep duration on weekdays. Other potential confounders such as physical activity level, extracurricular activities, socioeconomic status and substance use were not evaluated. Circadian rhythm markers of individual participants were not assessed but studies have shown that most children have a phase delay as they mature [18].

## Conclusions

In conclusion, our data suggests that children with newly diagnosed primary hypertension obtain less weekend catch-up sleep by waking up earlier on weekend. Hypertensive children have more blunted “subjective sleepiness” response to insufficient sleep. Future studies evaluating the relationship between sleep and BP should consider the effect of weekend sleep in addition to weekday sleep. Additional information will help in counseling our patients regarding weekend sleep patterns.

## Abbreviations

BMI-SDS: Body Mass Index-Standard Deviation Score
BP: Blood pressure
DBP: Diastolic blood pressure
HBP: High blood pressure
PDSS: Pediatric Daytime Sleepiness Scale
PLMS: Periodic leg movements of sleep
PSQ: Pediatric Sleep Questionnaire
SBP: Systolic blood pressure
SRBD: Sleep related breathing disorder
